# A systematic review of patient and healthcare professional perceptions of the barriers and facilitators to embedding exercise in the adjuvant cancer treatment pathway

**DOI:** 10.1007/s00520-026-10553-w

**Published:** 2026-03-18

**Authors:** Sarah Hodge, Jordan Curry, Julie Walabyeki, John M. Saxton, Victoria Brown, Maureen Twiddy

**Affiliations:** 1 Hull York Medical School, Institute of Clinical and Applied Health Research, University of Hull, Cottingham Road, Hull, UK; 2 Wolfson Palliative Care Research Centre, Hull York Medical School, University of Hull, Cottingham Road, Hull, UK; 3 Screening and Diagnostic Pathways Research Group, Hull York Medical School, Cancer Awareness, University of Hull, Cottingham Road, Hull, UK; 4 School of Sport, Exercise & Rehabilitation Sciences, University of Hull, Cottingham Road, Hull, UK; 5 Academic Department of Oncology, Queen’s Centre for Oncology and Haematology, Castle Hill Hospital, Cottingham, Hull UK

**Keywords:** Barriers, Cancer, Cancer patient, Chemotherapy, Exercise, Facilitators, Healthcare professional, Perceptions, Physical activity, Radiotherapy

## Abstract

**Purpose:**

Evidence suggests that physical activity and exercise interventions can mitigate cancer treatment side effects. However, an improved understanding of how physical activity/exercise can be embedded within cancer care pathways is needed. We examined what is known about the barriers and facilitators to implementing physical activity/exercise interventions in the adjuvant cancer treatment pathway from a patient and healthcare professional perspective. The protocol was registered a priori with PROSPERO in March 2023.

**Methods:**

Electronic databases (CINAHL Plus, MEDLINE, PsycINFO and Cochrane) were searched for quantitative, qualitative, and mixed methods evidence from 2004 to 2025. Quality appraisal was undertaken using the appropriate Critical Appraisal Skills Programme tools. Barriers and facilitators were inductively coded into themes and then mapped to the Capability-Opportunity-Motivation-Behaviour (COM-B) model and Theoretical Domains Framework (TDF). Thematic meta-synthesis was applied to the data.

**Results:**

Fifteen qualitative, twelve quantitative and two mixed methods studies met the inclusion criteria. Three core themes emerged that influenced implementation: exercise intervention, organisational setting and impact of cancer. The barriers and facilitators identified by patient and healthcare professional participants were relevant to all the COM-B constructs, with the most prevalent TDF domains being skills, knowledge, environmental context and resources, social influences and optimism.

**Conclusion:**

Findings emphasise knowledge and education, previous experience with exercise, resources, motivation, gender, age, social factors, positive promotion, individualised programming throughout treatment and access to credible practitioners as nuanced perspectives between patients and healthcare professionals. These converging perspectives are influential determinants to help identify potential solutions for embedding exercise within cancer treatment pathways.

**Supplementary Information:**

The online version contains supplementary material available at 10.1007/s00520-026-10553-w.

## Introduction

Globally, the number of new cancer diagnoses is projected to reach 28 million annually by 2040, with the UK ranking among the top 10% of countries in terms of incidence [1, 2]. It is proposed that roughly one in two individuals in the UK will experience a cancer diagnosis during their lifetime, with the most common types being breast, lung, prostate, and bowel [colorectal] [1, 3].

A range of treatment options exists for those living with cancer, including surgery, chemotherapy, radiotherapy, immunotherapy, hormone therapy, and targeted agents. Treatment approaches vary depending on cancer type, disease stage, tumour biology, characteristics, and the individual’s patient profile [4]. Where ‘curative intent’ treatments are not possible, other treatments are used to extend life and palliate symptoms through slowing down the growth of cancer cells [3, 4].

Despite advances in cancer treatment, both the disease and associated management can substantially impact health and wellbeing. Surgery may lead to nerve and muscle damage or scarring, affecting physical and psychological wellbeing [4]. Chemotherapy, which targets both healthy and cancerous cells, often leads to side effects such as infection, cardiotoxicity, nausea, vomiting, diarrhoea, and malnutrition [4]. Immunotherapy and targeted treatments may cause fatigue, skin changes, and digestive issues, while radiotherapy can impair swallowing and alter bladder or bowel function. Combination therapies, such as chemoimmunotherapy, can further intensify side effects and reduce quality of life [3, 4].

A growing body of evidence [3] suggests that adjunctive non-pharmacological interventions such as physical activity (PA) and/or exercise can mitigate and, in some cases, improve treatment side effects; however, definitions of what ‘PA’ and ‘exercise’ mean vary in literature, and these terms are frequently used synonymously. For this systematic review, ‘PA’ is defined as ‘any bodily movement produced by skeletal muscles that requires energy expenditure’ and ‘exercise’ as ‘any voluntary PA that is planned, structured, repetitive, and undertaken to sustain or improve health and fitness’, and is considered a subcategory of PA [5].

National and international guidance [5–9] recommends weekly, moderate intensity, vigorous and strength-based activity for adults, including those diagnosed with cancer. Although these recommendations appear feasible, empirical literature suggests that barriers exist to participation, particularly for cancer patients undergoing treatment. One systematic review [3] supports that PA/exercise may help reduce the physical and psychological symptoms associated with cancer treatment and preclinical and human intervention studies further support this idea [10–13].

**Fig. 1:**
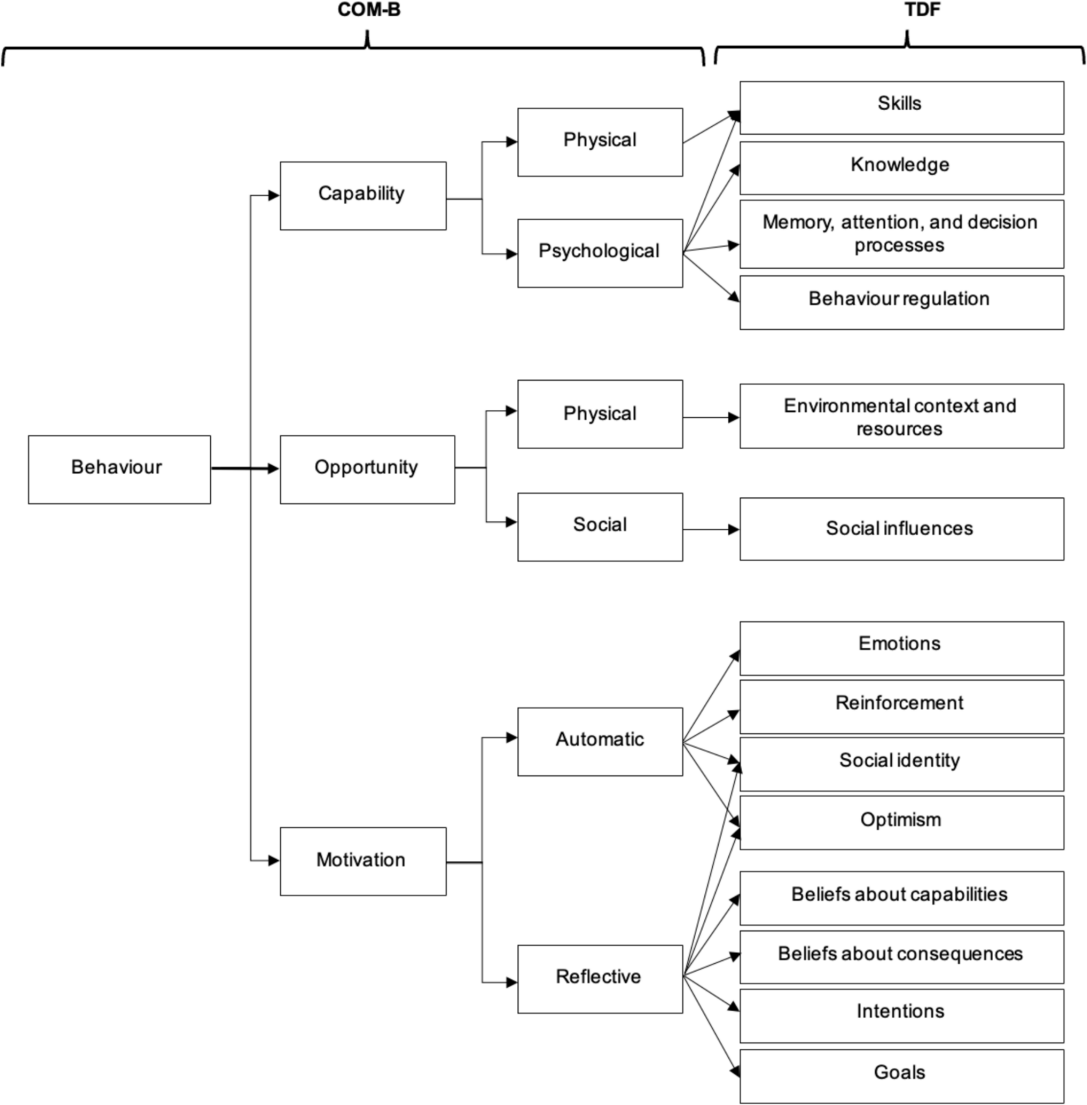
Capability, Opportunity, Motivation, Behaviour (COM-B Model) and Theoretical Domains Framework (TDF) Behaviour Change Domains adapted from [15]

This systematic review aimed to understand the barriers and facilitators of embedding PA/exercise in the adjuvant cancer treatment pathway from a patient and healthcare professional (HCP) perspective. To support this process, implementation science literature has been drawn upon with use of the Capability-Opportunity-Motivation-Behaviour (COM-B) model and Theoretical Domains Framework (TDF) [14] to present the determinants from patient and HCP perspectives to inform the direction of future research and assist in developing good practice implementation guidelines within cancer care. An outline of the COM-B model and TDF framework is presented in Fig. 1.

## Methods

The systematic review is reported according to the Preferred Reporting Items for Systematic Reviews and Meta-Analyses (PRISMA) guidelines [18] (see Online Resource 1). A protocol was registered with PROSPERO in March 2023 (ID: CRD42023394157).

### Search strategy

The Population, Exposure and Outcome (PEO) approach was employed to guide the development of search terms [16] (see Online Resource 2). Searches included Boolean operators to refine the search strategy. A search in March 2023 was conducted across 12 library and electronic databases (see Online Resource 2) from the years 2004 to 2024. An updated search was conducted in December 2024 and February 2026 across the same databases. Backwards citation searches were completed on both occasions using reference lists of the included studies.

### Study selection

All papers identified through the database searches were exported to EndNote (Version 20) for deduplication and imported to Covidence systematic review software (Veritas Health Innovation, Melbourne, Australia. Available at www.covidence.org). Papers were title and abstract screened against the eligibility criteria (see Table 1) independently by two authors (SH and JC). Conflicts were resolved through discussion and mutual consensus. Full-text screening was conducted by two authors (SH and JC), with conflicts resolved through discussion and mutual consensus, or with a third author (MT).

**Table 1:** Eligibility criteria

Inclusion	Exclusion
• Empirical studies	• Literature involving children/adolescents under the age of 18 years
• Literature involving adults aged 18 years and over	• Literature solely focusing on a specific exercise regime, e.g., upper limb rehabilitation/yoga
• Literature involving cancer patients	• Literature only involving cancer patients’ post-treatment/survivorship
• Literature involving healthcare professionals, exercise physiologists, exercise professionals	
• Literature focusing on exercise, general exercise interventions and physical activity	
• English language	
• Quantitative and/or Qualitative Methodology	

### Quality assessment of included studies

Quality appraisal was undertaken independently by authors SH, MT, JW, JS and VB with two authors reviewing each paper. The Critical Appraisal Skills Programme (CASP) tools for qualitative and quantitative (inclusive of randomised controlled trials) research [17] was adopted. Mixed methods papers were reviewed using both CASP tools to enhance rigour, reliability and validity [18–20]. Discussion took place after the quality appraisals were completed, and conflicts were mutually resolved. Where methodological flaws were identified in a study (see Online Resource 3), it was agreed that the papers would not be excluded if the study content highlighted potential gaps and valuable considerations to inform healthcare practice and future primary research. However, the limitations would be discussed in the review.

### Data extraction and data synthesis

A data extraction template was developed and piloted to ensure retrieval of information within the selected papers was relevant to the review question [21]. Methodological summary, participant characteristics, treatment type, outcome results and findings relevant to barriers and facilitators were extracted and recorded. Two separate data tables were generated to capture the extracted barriers and facilitators, including the frequency they are reported, from patients and HCPs.

Patient and HCP barriers and facilitators were reviewed separately. Coding was undertaken inductively to ensure that possible barriers were identified. These codes were then grouped into three core themes (Exercise Intervention, Setting and Impact of Cancer) and sub-themes as summarised (see Tables 2 and). The codes were then mapped to the COM-B and TDF to provide a broad multi-level description of the barriers and facilitators to help organise understanding of key determinants (see Online Resource 4 and Online Resource 5), how they crosscut and their impact on implementation. A thematic meta-synthesis approach [18] was used to bring the data together into a narrative [18] (Table 3).

**Table 2:** Patient themes and sub-themes after coding

Theme	Sub-theme	References	Description
**Patient barriers**			
Exercise intervention	Beliefs around capability	14, 26, 27, 29, 30, 35, 37, 42, 41, 49	Fear of participation and not being able to participate if no prior experience
	Social barriers and connection	24, 14, 27, 28, 33, 36, 46, 42, 48, 49	Impact of time, family roles and social events during treatment
	Lack of knowledge	24, 14, 23, 26, 27, 30, 35, 38, 46, 46, 47, 48	Highlights a lack of information provided and limited communication from HCPs
Setting	Environment	39, 24, 23, 26, 27, 28, 35, 37, 46, 45, 48, 49, 40	Considers the impact of location, time and access to facilities
	Organisational resources	24, 14, 30, 46, 42, 40	Identifies funding gaps and lack of equipment
	Perceptions of delivery	24, 14, 33, 46, 41, 47	Perceptions of environments that host physical activity and exercise and how demographic factors play into perceptions
Impact of cancer	Cancer site	14, 27, 33, 34, 36, 43, 46, 42	Highlights cancer site impact and specific associative symptoms
	Treatment related symptoms	39, 14, 23, 26, 27, 29, 30, 33, 35, 36, 38, 45, 43, 42, 41, 47, 40	Identifies the impact of different treatment types
	Emotional wellbeing and self-belief	24, 27, 28, 29, 30, 33, 36, 46, 42, 41, 47, 49	Identifies thoughts and feelings during cancer treatment
**Patient facilitators**			
Exercise intervention	Self-belief and capability	24, 14, 23, 26, 27, 37, 46, 42, 41, 47, 48	Identifies previous positive experiences with physical activity and exercise
	Positive communication	26, 28, 29, 30, 35, 36, 38, 37, 47, 48	Highlights the value of HCP and family encouragement
Setting	Individualised care	24, 23, 27, 28, 30, 33, 35, 37, 45, 41, 48, 40	Identifies importance of flexibility with availability and programming
	Social support and connection	24, 23, 28, 29, 30, 34, 35, 41, 48	Identifies group setting and support from family and others
	Credible sources	23, 26, 27, 28, 30, 34, 35, 36, 42, 41, 48	Identifies the confidence in having access to a specialist practitioner
	Accessibility and resources	24, 27, 30, 36, 41, 47	Highlights the impact of cost and location
Impact of cancer	Alleviate symptom burden	39, 23, 26, 27, 34, 35, 41, 48	Highlights specific facilities required within physical activity spaces
	Time-efficient pathway	23, 27, 33, 35, 46, 45, 47	Identifies when to approach physical activity and exercise

*Notes:HCP;* healthcare professional

**Table 3:** Healthcare professional (HCP) themes and sub-themes after coding

Theme	Sub-theme	References	Description
**HCP barriers**			
Exercise intervention	Beliefs and communication skills	25, 30, 31, 32, 44	Time and perceptions of patient ability
	Lack of knowledge	24, 22, 28, 30, 31, 44	Highlights limitations in resources, guidance, and associated concerns
Setting	Availability of resources	22, 25, 30, 31, 32, 44	Availability, space, and funding
	Lack of individualised care	39, 24, 27, 28, 30, 33, 35, 45, 41, 48	Highlights physical activity and exercise not being tailored to patient need
Impact of cancer	Symptom focus	22, 25, 31, 32	The medical focus on symptom management
	Belief about capability	22, 31, 32	Highlights thoughts around patient ability to participate in physical activity and exercise during cancer treatment
**HCP facilitators**			
Exercise intervention	Understanding patient capability	25, 30, 31, 32	The importance of understanding patient ability
	Training and education	22, 25, 30, 31, 32	Highlights communication, physical activity, and exercise as key areas for developing knowledge
Setting	Individualised care	22, 25, 28, 30, 31, 32, 44	The flexibility with resources and programming
	Sense of safety	22, 26, 34, 42	The value of teamwork and social influences
	Credible practitioner	25, 31	The value of access to a practitioner with specialist knowledge in physical activity and exercise
Impact of cancer	Culture shift	30, 31	The inclusion of physical activity in discussions
	Time-efficient pathway	25, 30, 31, 32	Highlights consideration of when to discuss physical activity, exercise, and referrals

## Results

**Fig. 2:**
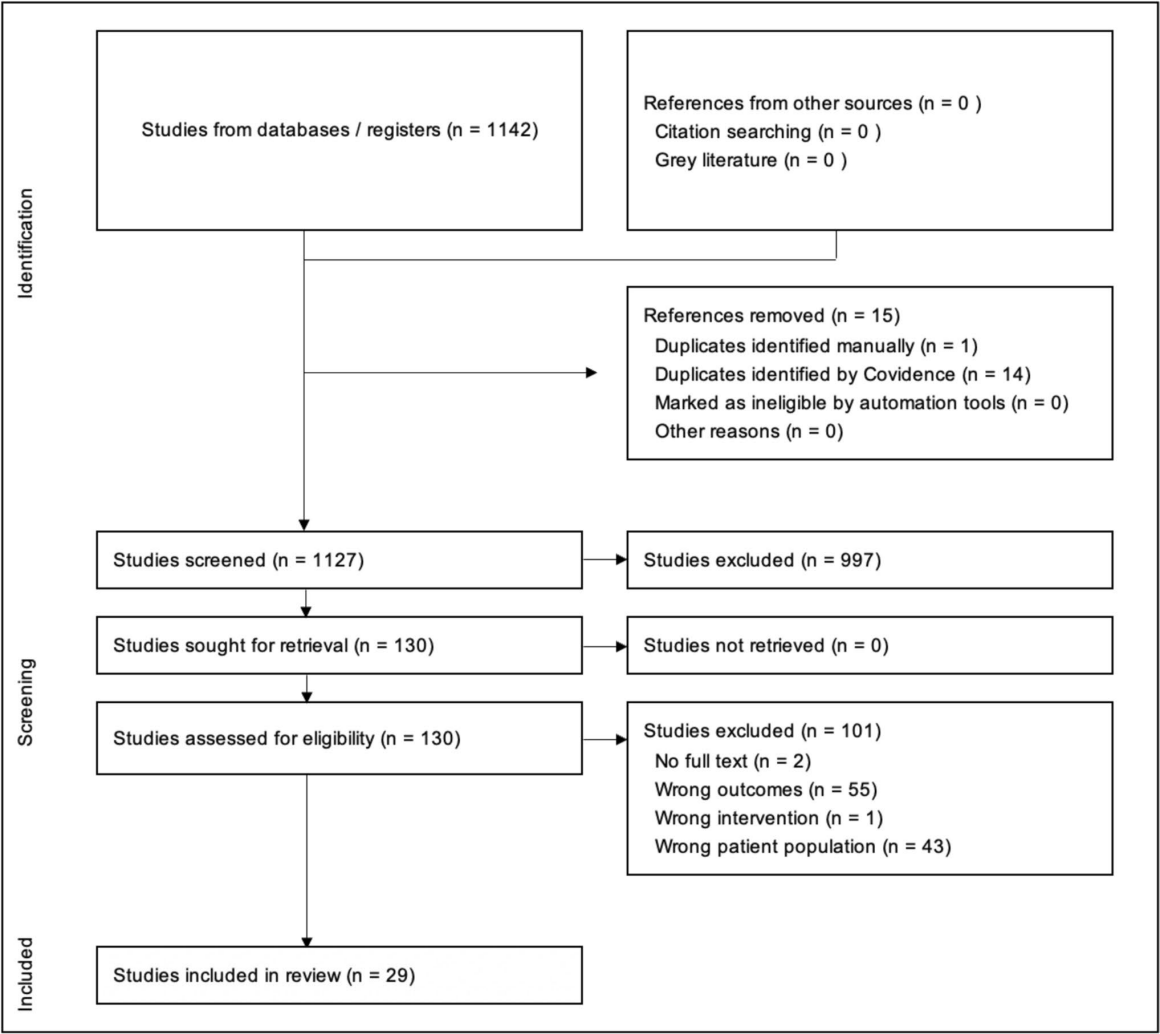
PRISMA figure [18]

The PRISMA flow chart is presented in Fig. 2. A total of 1142 publications were identified and following deduplication 1127 papers were title and abstract screened, of which 130 met the criteria for further review. The full texts of 130 papers were screened, resulting in 29 studies meeting the inclusion criteria.

### Study characteristics

This review included 15 qualitative studies [22–36], 12 quantitative [14, 27, 37–46] and three mixed methods studies [47–49].

All papers were deemed to have sufficient methodological quality for inclusion. One study was completed in Ireland [49], one in the Netherlands [24] and one in Switzerland [40], two each in Canada [39, 47], Italy [22, 23], Sweden [33, 48] and Denmark [14, 35], three in the UK [28, 30], four in the USA [26, 27, 38, 46], five in Australia [25, 31, 34, 43, 45] and six in Germany [32, 36, 37, 41, 42, 44].

The studies that included both patient and HCP perspectives were completed in the UK [28, 30]. Twelve studies incorporated multiple cancer types [14, 23, 24, 33, 35, 38, 40–42, 44, 45, 48] and other studies were specific to breast [27, 29, 30, 37, 39, 43, 46, 47], prostate [34, 36, 49], lung [26, 28, 31] and myeloma [27].

Of the studies involving patients, 17 incorporated multiple cancer treatment types [23, 24, 26, 29, 30, 33, 35–37, 40–44, 46–48] and seven focused on a single treatment arm such as surgery [28], chemotherapy [14, 38, 39], radiotherapy [45] or cancer site-specific treatment [27, 34, 49]. All patients were over the age of 18 years, with most patients aged over 50 years. Further information regarding the study characteristics can be found in Table 4.

**Table 4:** Characteristics of included studies (*n* = 29)

Study	Aim	Study design	Sample	Cancer type	Treatment type	Key findings
Avancini et al. (2021) [22]	To examine the perspectives of nurses about physical activity in cancer patients	Qualitative	14 healthcare professionals (Nurse)	Not specified	Not specified	Physical activity is an important strategy for cancer patients and nurses counselling may have a direct impact on this. Educational sessions dedicated to nurses to disseminate accurate evidence-based knowledge is necessary to improve their confidence when discussing physical activity with cancer patients
Avancini et al. (2020) [23]	To explore factors that influence an active lifestyle in cancer patients during treatment	Qualitative	18 patients	Colorectal, Pancreas, Ovary, Lung, Breast, Head and Neck, Melanoma, Thymus	Surgery, Chemotherapy, Radiotherapy, Hormone therapy, Immunotherapy, Target therapy	Physical activity is essential as a complementary tool for cancer patients. The barriers to physical activity are mainly associated with the cancer itself and treatment-related side effects
Bland et al. (2018) [37]	To identify the predictors of attendance to an oncologist-referred exercise program offered during adjuvant breast cancer treatment	Quantitative	68 patients	Breast	Chemotherapy, Radiotherapy	It is the identified barriers that are of interest to researchers and healthcare providers. There is a strong association between quality of life, income, marital status and post-surgery treatment with attendance on exercise programs
Caperchione et al. (2021) [25]	To understand factors that impact implementation of exercise communication and referral and explore integrated clinical approaches to exercise communication and referral in cancer care	Qualitative	53 healthcare professionals [Breast cancer care nurse, Exercise physiologist, Physiotherapist, Radiation oncologist, Medical oncologist, Cancer care nurse, Social worker, Wellness manager, Haematologist)	Not specified	Not specified	The study proposed a model/outline of a referral pathway that may guide implementation of an exercise referral program into medical settings. This included oncologist-initiated communication, resources and access to exercise professionals with cancer expertise The testing of efficacy of the pathway in a ‘real world’ clinical setting is required to aid understanding around the practical and logistical implications of the proposed pathway
Cheville et al. (2012) [26]	To describe the beliefs of cancer patients with late-stage disease regarding exercise	Qualitative	20 patients	Lung	Chemotherapy, Radiotherapy	For exercise to be effective to reduce cancer-related symptoms there needs to be understanding of the patient’s usual and past activities (e.g., level of exercise), proactive negotiation of potential barriers, education and the positive support of their oncologist
Clark et al. (2007) [38]	To assess the variables related to self-efficacy for increasing participation in physical activity in advanced-stage cancer patients receiving chemotherapy	Quantitative	128 patients	Breast, Digestive, Lung, Gynaecological, Genitourinary, Neurologic, Head and Neck, Other	Chemotherapy	Patients with advanced-stage cancer who receive chemotherapy are interested in maintaining or increasing their physical activity level. Physical activity interventions should include tailored information and discussion regarding the specific benefits from a professional. Barriers to physical activity must be considered yet the physical activity itself could help overcome these, e.g., managing fatigue
Coon and Coleman (2004) [27]	To learn about the feelings, beliefs and experiences of patients implementing an exercise program in the context of a specific protocol	Quantitative	21 patients	Multiple Myeloma	Chemotherapy	The interaction between patients’ beliefs, social context and previous experience had the potential to have a positive or negative influence on their ability to adhere to an exercise program during treatment
Courneya et al. (2008) [39]	To report the barriers to supervised exercise in breast cancer patients	Quantitative	242 patients	Breast	Chemotherapy	Barriers to supervised exercise vary. The majority of barriers are associated with the cancer itself and treatment. Conclusion was that behavioural support programmes should focus on strategies to maintain exercise when treatment side effects are difficult
Crandall et al. (2018) [28]	To explore the views, attitudes and beliefs of key stakeholders on exercise intervention for people who are surgically treated for lung cancer	Qualitative	12 patients and 11 healthcare professionals (Physiotherapist, Clinical Nurse Specialist, Chest Physician)	Lung	Surgery	Barriers to exercise appear to be linked to personal factors, environment, and resources. The study emphasises the importance of including key stakeholders, such as HCP’s, in the development of exercise interventions for patients
Del Arco et al. (2025) [40]	To compare oncologists, physical activity recommendations, physical activity patterns and barriers in two different geographical areas	Quantitative	254 patients	Not specified	Not specified	Physical activity patterns seem to differ according to the patient’s country. Physical activity should be carried out taking into account sociodemographic, cultural, climatic and structural characteristics in order to be personalised
Emslie et al. (2007) [29]	To explore the experience of women undergoing breast cancer treatment who had taken part in a supervised group exercise trial	Qualitative	36 patients	Breast	Chemotherapy, Radiotherapy, Chemotherapy and Radiotherapy	Barriers to physical activity can be overcome through using a gender-sensitive approach and providing a supportive group environment
Felser et al. (2019) [41]	To investigate the knowledge, experience, motivation to participate in exercise and the preferences of cancer patients to participate in exercise groups	Quantitative	172 patients	Lymphoma, Leukaemia, Urogenital, Gastrointestinal, Pulmonary, Other	Surgery, Chemotherapy, Surgery and Chemotherapy, Chemotherapy and Radiotherapy, Surgery and Chemotherapy and Radiotherapy, Other	Exercise participation depends on physical training experiences before cancer and during treatment. The motivation of cancer patients for physical exercise might be increased with the integration of therapy-accompanied exercise programmes
Fernandez et al. (2015) [47]	To explore the barriers and facilitators to exercise in individuals with cancer	Mixed Methods	30 patients	Breast	Surgery, Chemotherapy	The study concluded that patients exercise levels decrease when undergoing cancer treatment. Symptoms from the cancer and/or treatment were the main barriers and more thorough and consistent education is needed as to why exercise is beneficial during treatment
Frikkel et al. (2020) [42]	To identify the barriers and motivation to physical activity in advanced cancer patients with tiredness/weakness	Quantitative	141 patients	Gastrointestinal, Lung, Breast, Sarcoma, Other (Genitourinary, Gynaecological, Cancer of Unknown Primary, Glioblastoma, Others)	Chemotherapy, Immunotherapy, Antibody Therapy (combined with hormonal treatment or monotherapy), Targeted Therapy (combined with hormonal treatment or monotherapy), Hormonal treatment	The positive motivation towards physical activity is the strongest predictor of behaviour. Interdisciplinary programmes may be beneficial to support motivation and participation in physical activity
Gho et al. (2013) [43]	To determine the relationship between patient characteristics, physical side effects, exercise bra discomfort and exercise behaviours	Quantitative	432 patients	Breast	Mastectomy or Lumpectomy then Chemotherapy	Exercise bra discomfort was positively associated with not achieving the recommended levels of exercise. The physical side effects associated with breast cancer surgery are also a contributing factor to reduced participation in physical activity/exercise
Gokal et al. (2024) [30]	To explore patient and HCP views about integrating conversations and support for physical activity into routine care during treatment for breast cancer	Qualitative	15 patients and 11 healthcare professionals (medical, nursing)	Breast	Chemotherapy	HCPs are reluctant to discuss the topic of participation in physical activity, yet patients would welcome discussions. Educating patients about physical activity reducing the risk of recurrence along with evidence-based, low-cost remote interventions would allow HCPs to integrate conversation about physical activity within routine cancer care for patients
Granger et al. (2016) [31]	To identify barriers and facilitators that influence clinicians’ translation of the physical activity guidelines into practice	Qualitative	17 healthcare professionals (Medical Doctor, Nurse, Physical therapist working in lung cancer)	Lung	Not applicable	Barriers to promoting physical exercise were found at patient, clinician and healthcare system level
Haussman et al. (2018) [32]	To describe the influencing factors for HCP’s physical activity promotion behaviour and the mechanisms behind them	Qualitative	30 healthcare professionals (GPs, Specialist Physicians, Oncology Nurses)	Breast, Prostate, Colon	Chemotherapy, Radiotherapy, Surgery	HCPs workload impacted on referral of patients into exercise programmes. In-house professional to support this process would be beneficial. Structural factors such as length of consultation time or availability of exercise programmes would have the greatest impact on promoting physical activity
Haussman et al. (2018) [44]	To examine how structural barriers are associated with promoting physical activity and how HCPs react to information resources	Quantitative	917 healthcare professionals (GP, Medical Oncologist, Radiation Oncologist, Gastroenterologist, Urologist, Gynaecologist, Surgeon, Other Medical Specialities)	Breast, Prostate, Colorectal, Lung, Other	Chemotherapy, Radiotherapy, Surgery, Aftercare, Other	HCPs [physicians] in inpatient care would particularly benefit from an expert contact person in relation to physical activity/exercise. For nurses working in outpatient services, more time would be beneficial. All HCP subgroups indicated their interest in information resources suggesting physical activity for cancer patients is recognised as beneficial
Henriksson et al. (2016) [33]	To describe cancer patients perceived barriers and facilitators of physical activity during adjuvant cancer treatment	Qualitative	23 patients	Breast, Prostate, Colorectal	Radiotherapy and Endocrine Therapy, Chemotherapy, Endocrine Therapy	Side-effects from treatment were the main barriers. It was suggested that information about physical activity from HCPs should be given early after diagnosis and as part of standard care
Ijsbrandy et al. (2019) [24]	To identify patients’ experienced barriers and facilitators in implementing physical activity programs for patients with cancer	Qualitative	34 patients	Breast, Abdominal, Pelvic, Haematological, Bone, Lung	Surgery, Chemotherapy, Radiotherapy, Hormonal Therapy	A multifaceted strategy focusing on different domains of barriers would be more successful in implementation in healthcare systems because barriers often arise at different levels. All barriers affect implementation, and any one barrier affects others therefore testing multifaceted strategies would be reasonable
Keogh et al. (2014) [34]	To examine the perceptions of men with prostate cancer of their barriers and facilitators to physical activity and how treatment may influence their perceptions	Qualitative	14 patients	Prostate	Androgen Deprivation Therapy (ADT)	Clinicians should frequently discuss how physical activity is beneficial for prostate cancer patients. Spouses and/or close family and friends are potentially the main facilitators for patients to participate in physical activity
Mazzoni et al. (2019) [48]	To explore the motivational experiences of exercise combined with behaviour change support	Mixed methods	229 patients	Breast, Colorectal, Prostate	Chemotherapy, Radiotherapy, Endocrine	Incentives and creating an environment that fosters feelings of autonomy, competence and relatedness will help encourage patients to participate in exercise during oncological treatment. Social support, structuring the physical environment and self-monitoring tools can be affective for adherence
Midtgaard et al. (2009) [14]	To investigate self-reported physical activity behaviour, exercise motivation and information in cancer patients undergoing chemotherapy	Quantitative	451 patients	Gynaecological, Breast, Colon, Testis, Oesophagus, Brain, Pharynx, Pancreas, Stomach, Haematological Malignancies	Chemotherapy	Patients with cancer undergoing treatment will be more motivated to participate if they are provided with information, advice and support regarding exercise and exercise programming whilst undergoing treatment
Mikkelson et al. (2019) [35]	To explore attitudes towards physical activity and exercise among older patients with cancer to inform future exercise-based interventions	Qualitative	23 patients	Pancreatic, Biliary Tract or Non-Small Cell Lung Cancer	Palliation (first line palliative chemotherapy or Immunotherapy)	Improving wellbeing and quality of life, fixed conditions, social support and familiar activities were identified as motivators and facilitators for physical activity
Murnane et al. (2010) [45]	To compare pre-treatment versus on-treatment activity levels of outpatients receiving radiotherapy and to identify patients’ preferences and barriers to exercise during this time	Quantitative	92 patients	Head and neck, Prostate, Breast, Gastrointestinal, Gynaecological, Haematological, Lung, Melanoma, Other	Radiotherapy and Chemotherapy, Radiotherapy	Learning about the benefits of exercise throughout cancer treatment is likely to increase the understanding and motivation of patients to remain or become physically active during cancer treatment
Murphy et al. (2024) [49]	To inform acceptability of a 6-month supervised intervention that emphasised increasing and varied intensities of aerobic exercise by exploring the experiences of men	Qualitative	12 patients	Prostate	Androgen Deprivation Therapy (ADT), Radiotherapy	Lack of self-confidence can be a barrier to exercise participation however exercise programmes have the potential to provide psychosocial benefits, rebuild confidence and empower men throughout their cancer treatment. Trust building, flexible delivery and credibility alongside a challenging exercise prescription are important facilitators for acceptability
Rogers et al. (2007)[46]	To determine the exercise barriers, outcome expectations/values and associations with exercise stage of change and exercise preferences	Quantitative	23 patients	Breast	Chemotherapy, Hormonal Therapy, Chemotherapy and Hormonal Therapy	Common exercise adherence barriers including lack of priority, self-discipline, procrastination and fatigue. Strategies to embed exercise from the point of diagnosis throughout this specific treatment should reflect men’s experiences of exercise
Sheill et al. (2018) [36]	To examine the attitudes of patients living with metastatic prostate cancer towards physical activity	Qualitative	20 patients	Prostate (Metastatic)	Radiotherapy, Hormonal Therapy	There is a need to increase prompts that encourage patients with metastatic cancer to maintain/increase their physical activity level post diagnosis. Patients have individualised needs, and a facilitator would be a referral to a cancer exercise specialist for the prescription of tailored physical activity programmes per patient

*Notes: HCP;* healthcare professional

## Barriers and facilitators—overview

The barriers and facilitators identified by patient and HCP participants were relevant to all COM-B constructs and 11 of the 14 TDF domains including skills, knowledge, behaviour regulation, environmental context and resources, social influences, emotions, social identity, optimism, beliefs about capabilities, beliefs about consequences and goals.

### Patient barriers

#### Capability-related barriers

Patients reported a lack of information regarding PA being communicated to them via HCPs during their cancer treatment [14, 23, 24, 30, 47] and a lack of ‘qualified persons’, e.g., an exercise physiologist or physiotherapist, available to provide guidance around PA [24, 49]. The impact of cancer and physical treatment side effects [14, 23, 26–28, 36, 39, 41, 43] such as fatigue [14, 27, 32, 36, 39, 40, 42, 45], pain [14, 43] nausea [14, 27] and vomiting [27, 41] were frequently reported by patients as a barrier during chemotherapy [14, 27, 42, 47]. Patients reported disease and progression [27, 36] and specific psychological symptoms associated with their cancer site and treatment, e.g., body image [29] and highlighted the impact that treatment has in parallel to their pre-existing co-morbidities [35] such as physical limitations from musculoskeletal conditions like osteoarthritis.

#### Opportunity-related barriers

Patient studies outside of the UK reported better insurance cover could improve waiting times and access to PA/exercise, e.g., more accessible locations at more convenient times [14, 24, 40, 46]. This was corroborated by HCPs [22, 24, 28, 30–32, 44] across both UK-based and international studies. From a social perspective, female patients with care responsibilities identified family commitments as a barrier due to the time spent on fulfilling their parental role [14, 27, 28, 35, 42, 46, 48] or caring for elderly relatives. Patients also reported difficulty engaging in exercise independently without knowing a safe benchmark of what ‘other patients’ were doing during treatment [14, 33, 36, 49] and yet feeling ‘vulnerable’ in group settings due to reduced confidence or concerns about contracting an infection [24, 33, 49].

#### Motivation-related barriers

Patients frequently reported they perceived PA/exercise to be ‘harmful’ and expressed a ‘fear’ of worsening their cancer and symptoms during treatment [14, 26–28, 30, 35, 49]. Older patients reported ‘age’ as a barrier [1, 33, 41, 45] and perceptions that exercise spaces are for ‘younger people’ only, resulting in demotivation to participate. Generally, patients reported reduced self-confidence during cancer treatment [24, 28–30, 37, 41, 47, 49] with frequent reporting of low mood and motivation associated with body image post-surgery in breast, colorectal and prostate cancer patients, or the reduced feeling of ‘masculinity’ [28, 33, 36, 42, 46] within prostate cancer patients. These barriers reportedly had an impact on a patient’s ‘readiness’ and ‘interest level’ [27, 42, 46, 47] to participate in PA/exercise. Lack of enjoyment in the exercises provided was also reported particularly from breast and prostate cancer patients [46, 48] whilst undergoing treatment.

### Healthcare professional barriers

#### Capability-related barriers

HCPs reported a lack of written resources on PA/exercise for medical staff to deliver or provide for patients with cancer [22, 24, 28, 30–32, 44]. HCPs reported consultations as ‘time-limited’ [44] therefore, discussions around exercise and PA are only prompted when patients asked about it [25, 30]. Communication skills in behaviour change were viewed as necessary when speaking with patients about PA/exercise [25, 30–32].

#### Opportunity-related barriers

Similar to patients, HCPs across medical and nursing disciplines frequently reported a lack of exercise programme availability for them to refer a patient into [22, 24, 28, 30–32, 44], limited capacity for exercise provision within hospital settings [25, 31] and lack of ‘time’ during their shift or consultation to promote PA and exercise [22, 30, 31] across various healthcare systems. This was a less common perspective amongst allied health professionals and exercise physiologists.

#### Motivation-related barriers

HCPs reported a lack of drive to discuss PA or exercise with a patient through fear of causing distress or harm to them if they are too unwell to participate [22, 31, 32]. If patient symptoms were particularly prominent during a consultation, e.g., pain or fatigue, HCPs were less likely to discuss exercise although this varied across cancer sites [25, 31], with it most commonly reported in the context of lung cancer.

### Patient facilitators

#### Capability-related facilitators

Patients frequently reported previous positive experience with exercise prior to commencing treatment as a facilitator for embedding PA/exercise into their daily routine whilst undergoing treatment [23, 26, 27, 35, 41, 42, 47].

#### Opportunity-related facilitators

Patients reported that when suitable exercise opportunities were available close to home or costs to attend or travel were low, they were more likely to engage in it [24, 30, 36, 40, 41]. Also, accessible toilet facilities were highlighted as crucial when attending exercise classes [41] for patients with colorectal or prostate cancer who experienced difficulties with continence or had continence appliances in situ [36]. Patients reported individualised programming [27, 28, 30, 33, 35, 41, 45, 48], flexible training times [30, 37, 41] and the opportunity to self-monitor progress would be beneficial. Older patients reported they found value in social support and connection in the form of group exercise sessions [30, 35, 41, 48], family involvement [23, 28, 34] and friendships [23, 24, 28, 29, 34] as motivators for consistently participating in PA during treatment.

The opportunity to participate in research associated with exercise was reported by patients as a facilitator [27] with positive promotion from family and HCPs [26, 29, 30, 35–38, 42, 47, 48], the offer of an exercise prescription [37, 40, 47] and positive participation experiences [23, 26, 27, 35, 41, 42, 47] as potentially key determinants for embedding exercise into practice.

#### Motivation-related facilitators

Regardless of previous exercise experience, patients across age ranges and treatment stages reported their motivation to participate would be enhanced if it was proven to benefit their quality of life [14, 24, 26, 27, 37, 41, 42, 48]. Patients across gender groups also reported that if exercise was individualised [27–30, 33, 35, 41, 45, 48] and programmed throughout their treatment plan [23, 27, 33–35, 45–47], they would feel more motivated to participate, particularly if it had been recommended by a HCP. Support from family [26, 29, 30, 35–38, 42, 47, 48] and a credible exercise practitioner leading sessions [23, 26, 28, 30, 35, 36, 41, 48] were also identified here as motivators to attendance and ongoing participation during treatment.

### Healthcare professional facilitators

#### Capability-related facilitators

Medical HCPs reported the key things that supported them discussing PA/exercise with patients was having education resources and guidance that they could provide for patients [22, 25, 30–32], receiving training on communication skills in relation to behaviour change [22, 25, 30] and prioritising PA in consultations [30, 31]. Generally, across all HCP disciplines it was reported that for some patients’ PA and exercise was a form of ‘escapism’ from their treatment [30] which enabled HCPs to feel more capable of discussing it. HCPs reported they were less likely to recommend PA/exercise if they felt a patient lacked the capability, e.g., less likely to communicate PA/exercise to female patients based on their perception of them having more caregiving roles and subsequent lack of availability to participate [32, 44] which was echoed in female patient perspectives.

#### Opportunity-related facilitators

Specialist knowledge and presence from a credible exercise practitioner [23, 26, 28, 30, 35, 41, 48] and guidance from a medical professional [26, 34, 42] were considered facilitators by medical HCPs [25, 31] but not nursing or allied health professionals (AHPs). Additionally, the opportunity for a ‘teamwork’ approach between the patient, their family and HCP [22] was reported as having the potential to evoke a sense of safety and support for patients who lack confidence.

For patients receiving cancer treatment, medical HCP groups generally reported the delivery of information about PA/exercise needs to be compatible with treatment stage [25, 30–32] e.g., it may not be prudent to discuss exercise in the palliative phase of treatment due to deterioration in symptoms. To overcome this, it is indicated across both UK and international studies that conversations about this topic should be frequent throughout the treatment pathway [30, 31] and the opportunity to directly refer patients for PA/exercise any time that was appropriate during their cancer journey as opposed to being GP led [25, 31] is important.

#### Motivation-related facilitators

HCPs reported an understanding of the patient’s psychological wellbeing, referred to as ‘headspace’, or their perceived ‘readiness’ would determine whether they would discuss PA/exercise with a patient [25, 30, 32]. Furthermore, they reported being more optimistic about discussing PA/exercise if they felt the patient was physically able, or appeared interested, e.g., if a patient reported positive experiences with exercise prior to treatment [25, 30, 32]. Additionally, directly witnessing a patient’s positive physical or emotional response to exercise participation during treatment [25, 31] would act as a motivator for consistent discussion.

## Discussion

### Summary of main findings

**Fig. 3:**
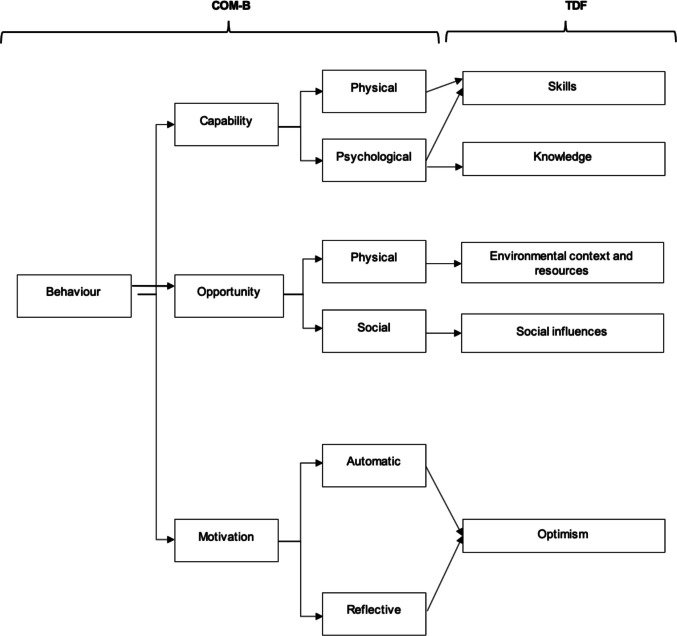
Capability, Opportunity, Motivation, Behaviour (COM-B Model) and Theoretical Domains Framework (TDF) Behaviour Change Domains adapted from [15]

The systematic review aimed to explore patient and HCP perceptions regarding the barriers and facilitators to embedding exercise into the adjuvant cancer treatment pathway with a view to informing implementation within healthcare services and future research. Generally, across UK-based and international studies PA/exercise in the cancer pathway was viewed positively and there was overlap of perspectives from both patients and HCPs across five of the 14 TDF domains including skills, knowledge, environmental context and resources, social influences and optimism as presented in Fig. 3. Therefore, the focus in this discussion is on these converging perspectives as the concordance between them enhances the power and potential impact of the identified barriers.

A large, recent, systematic rapid review of 118 studies [50] concluded that having access to appropriate advice and written information about PA/exercise and available services would overcome barriers such as lack of skills or knowledge across both patient and HCPs. However, most of the included studies within the review focused on the post-treatment phase, with the suggestion that cancer patients should aim to increase PA/exercise participation only as opposed to considering implementation within healthcare pathways.

Most reviews have limited generalisability as the data captured is only obtained from cancer survivors [51, 52] therefore difficult to ascertain if these perceptions are reflective of patients undergoing treatment, some of whom will not survive their treatment. In the present review, the use of COM-B and TDF reiterated that patients and HCPs alike reported systemic barriers within the capability domain such as having little access to information in relation to the value, safe parameters or recommendations for PA/exercise during treatment. This consequently had a negative impact on the patient’s confidence to participate in PA/exercise and/or for HCPs knowledge to discuss it during clinic appointments.

Within the opportunity domain, both patients and HCPs agree that individualised programming is essential, be that a prescription or compatibility with stage of treatment [27, 28, 30, 33, 35, 41, 45, 48] and both groups report that access to a skilled exercise professional is important [23, 26, 28, 30, 35, 36, 41, 48] which is also reflected in earlier literature [50–52]. Our review found that HCPs generally support this view; oncologists and nursing staff were more tentative and saw the need to have access to suitable exercise interventions for different cancers and/or witness the benefits of exercise for patients, e.g., receiving positive feedback or observing an improvement in symptoms [39], before feeling confident about actively promoting exercise it within consultations [25, 30, 32]. This suggests that access to resources and education may not be adequate without positive lived experience of PA/exercise participation, which highlights the value of acknowledging the overlap of determinants when considering implementation planning.

Use of the COM-B and TDF within this review presents motivation as a determinant to consider across different cancer sites and reports how some barriers may be disease specific such as breast cancer, e.g., female patients lacking self-confidence to attend group exercise programmes due to post-surgical scarring and altered body image [29]. Where other reviews have focused on a gender specific cancer site, this review is the first to recognise ‘gender’ as a potential barrier and highlights gendered disparity in both patient-reported barriers and HCP communication, with female patients more often facing challenges to exercise participation and receiving less engagement on the topic from clinicians [14, 27–29, 35, 42, 46, 48, 49]. This highlights an important consideration around the impact of ‘social influences’ and perspectives for the equitable integration of exercise into cancer care pathways.

## Strengths of the review

The review has complied with PRISMA reporting standards and no major methodological flaws were identified within the included studies. This is the first systematic review that draws upon implementation science literature and adopts combined use of COM-B, TDF and thematic meta-synthesis to explore the barriers and facilitators of embedding exercise across various cancer sites for adults undergoing cancer treatment. Furthermore, it includes a scope of studies across all methodological approaches and both patient and HCP perspectives across secondary care disciplines in nuance.

Previous literature which applied the COM-B and TDF frameworks [51] presented the constructs in a segregated manner. However, in the present review, the application of thematic meta-synthesis has provided further insights by highlighting the intersectionality of patient and HCP perspectives which could guide focus when implementation planning. Additionally, this review shows that barriers within the capability and motivation domains are often intertwined. For example, patients reported treatment side effects as having an impact on their belief about their capability and motivation to participate. If patients present these thoughts in consultations, HCPs reported that they were less likely to speak about PA/exercise which demonstrates one of several crosscuts.

## Limitations of the review

Some of the included studies focused on a specific cancer site, e.g,. breast or prostate cancer or specific treatment, which provides an inward focus on specific areas. Nevertheless, these papers have helped identify clear barriers and facilitators that, as demonstrated in this review, echo across other cancer sites and therefore need to be considered when embedding PA/exercise within care pathways.

It is important to acknowledge that all studies were written in English, and not all studies were UK-based nor inclusive of underserved communities, making it difficult to generalise findings across different communities and/or healthcare systems which would influence the relevance of the organisational and structural barriers identified. Furthermore, little insight has been gained about how those HCPs responsible for the commissioning of services see the reported issues, to understand the higher-level barriers to successful implementation of PA/exercise within cancer care.

## Implications for clinical practice and future research

Previous research has considered the impact of multi-faceted strategies to PA/exercise implementation as barriers arise at different levels in a healthcare system and evidence suggests that any one barrier can influence another [24]. Patients frequently share their personal experiences such as the psychological impact of PA/exercise and list barriers and facilitators as individual factors without relating them to one another, whereas HCPs perceive the wider external issues such as logistics and organisational challenges. This demonstrates the breadth of perspectives that can be obtained through using both participant types in future research. This review also highlights some converging perspectives between patients and HCPs, including the need for knowledge and education [14, 22–24, 30–32, 44, 47], previous positive experience with exercise [14, 23, 26, 27, 41, 42, 47], resources [30, 31, 44], motivation [24, 27, 34, 41, 42, 48], gender [14, 29, 44], age [33, 35, 41], social factors [23, 24, 28–30, 34, 35, 40, 41, 48], positive promotion [26, 29, 30, 35–38, 42, 47, 48], individualised exercise programming throughout treatment [27, 28, 30, 33, 35, 40, 41, 45, 48] and access to a credible practitioner [23, 26, 28, 30, 35, 36, 41, 48]. These crosscuts are recognised as facilitators to potentially prioritise when developing services and go some way to exploring possibilities for how PA/exercise support could be provided within NHS cancer care.

## Conclusion

Despite the guidance and recommendations regarding the benefits of PA/exercise for patients undergoing cancer treatment, there continue to be barriers around its implementation, as insight into the challenges faced and how best to integrate PA/exercise interventions into cancer care is needed. Our findings emphasise the nuances between patient and HCP perspectives, with points of convergence helping to understand the influences on chosen behaviours towards exercise, as well as identify and understand potential solutions and feasibility from both perspectives for what may be required from a wider organisational perspective when considering changes to healthcare services.

## Supplementary Information

Below is the link to the electronic supplementary material.

ESM 1 (DOCX 21.6 KB)

ESM 2 (DOCX 15.7 KB)

ESM 3 (DOCX 20.2 KB)

ESM 4 (DOCX 21.4 KB)

ESM 5 (DOCX 17.9 KB)

## Data Availability

No datasets were generated or analysed during the current study.

## References

[1] What is cancer? [Internet]. UK: Cancer Research UK; 2023 [cited 2026 Mar 12]. Available from: https://www.cancerresearchuk.org/about-cancer/what-is-cancer

[2] Doughty HC, Hill RA, Riley A, Midgley AW, Patterson JM, Boddy LM et al (2023) Barriers to and facilitators of physical activity in adults living with and beyond cancer, with special emphasis on head and neck cancer: a systematic review of qualitative and mixed methods studies. Support Care Cancer 31(8):471 37458858 10.1007/s00520-023-07925-x PMC10352410

[3] Mishra SI, Scherer RW, Snyder C, Geigle PM, Berlanstein DR, Topaloglu O (2012) Exercise interventions on health‐related quality of life for people with cancer during active treatment. Cochrane Database of Systematic Reviews (8) 10.1002/14651858.CD008465.pub2 PMC7389071 22895974

[4] Physical activity: brief advice for adults in primary care [Internet]. UK: National Institute for Health and Clinical Excellence; 2013 [cited 2026 Mar 12]. Available from: https://www.nice.org.uk/guidance/ph44/chapter/2-Public-health-need-and-practice

[5] Caspersen CJ, Powell KE, Christenson GM (1985) Physical activity, exercise, and physical fitness: definitions and distinctions for health-related research. Public Health Rep 100(2):126–131 3920711 PMC1424733

[6] American Cancer Society Guideline for Diet and Physical Activity [Internet]. USA: American Cancer Society; 2025 [cited 2026 Mar 12]. Available from: https://www.cancer.org/healthy/eat-healthy-get-active/acs-guidelines-nutrition-physical-activity-cancer-prevention/guidelines.html

[7] Physical activity guidelines for cancer [Internet]. USA: American College of Sports Medicine; 2019 [cited 2026 Mar 12]. Available from: https://acsm.org/physical-activity-guidelines-cancer-infographic/

[8] Noyes J, Booth A, Flemming K, Garside R, Harden A, Lewin S et al (2018) Cochrane qualitative and implementation methods group guidance series—paper 3: methods for assessing methodological limitations, data extraction and synthesis, and confidence in synthesized qualitative findings. J Clin Epidemiol 97:49–58 29247700 10.1016/j.jclinepi.2017.06.020

[9] Yang L, Morielli AR, Heer E, Kirkham AA, Cheung WY, Usmani N et al (2021) Effects of exercise on cancer treatment efficacy: a systematic review of preclinical and clinical studies. Cancer Res 81(19):4889–4895 34215623 10.1158/0008-5472.CAN-21-1258 PMC9397632

[10] Ashcraft KA, Warner AB, Jones LW, Dewhirst MW (2019) Exercise as adjunct therapy in cancer. Semin Radiat Oncol 29(1):16–24 30573180 10.1016/j.semradonc.2018.10.001 PMC6656408

[11] Elise P, Caty G, Aboubakar Nana F, Reychler G (2020) Effects of exercise therapy in cancer patients undergoing radiotherapy treatment: a narrative review. SAGE Open Med 8:205031212092265 10.1177/2050312120922657 PMC7301662 32595968

[12] Lavallée JF, Abdin S, Faulkner J, Husted M (2019) Barriers and facilitators to participating in physical activity for adults with breast cancer receiving adjuvant treatment: a qualitative metasynthesis. Psychooncology 28(3):468–476 30657225 10.1002/pon.4980

[13] Schmitz KH, Potiaumpai M, Schleicher EA, Wolf LJ, Doerksen SE, Drabick JJ et al (2021) The exercise in all chemotherapy trial. Cancer 127(9):1507–1516 33332587 10.1002/cncr.33390

[14] Midtgaard J, Baadsgaard MT, Møller T, Rasmussen B, Quist M, Andersen C et al (2009) Self-reported physical activity behaviour; exercise motivation and information among Danish adult cancer patients undergoing chemotherapy. Eur J Oncol Nurs 13(2):116–121 19230768 10.1016/j.ejon.2009.01.006

[15] Michie S, van Stralen MM, West R (2011) The behaviour change wheel: a new method for characterising and designing behaviour change interventions. Implement Sci 6:42 21513547 10.1186/1748-5908-6-42 PMC3096582

[16] Boland ACG, Dickson R. Doing a systematic review. London: Sage; 2017.

[17] Long HA, French DP, Brooks JM (2020) Optimising the value of the critical appraisal skills programme [CASP] tool for quality appraisal in qualitative evidence synthesis. Res Methods Med Health Sci 1(1):31–42

[18] Hannes K, Macaitis K (2012) A move to more systematic and transparent approaches in qualitative evidence synthesis: update on a review of published papers. Qual Res 12(4):402–442

[19] Higgins J GS (2008) Cochrane handbook for systematic reviews of interventions: Cochrane Database Systematic Reviews, p 187–235

[20] Page MJ, McKenzie JE, Bossuyt PM, Boutron I, Hoffmann TC, Mulrow CD et al (2021) The PRISMA 2020 statement: an updated guideline for reporting systematic reviews. BMJ 372:n71 33782057 10.1136/bmj.n71 PMC8005924

[21] Data collection form [Internet]. UK: Cochrane Effective Practice and Organisation of Care (EPOC); 2017 [cited 2026 Mar 12]. Available from: https://www.google.com/url?sa=t&rct=j&q=&esrc=s&source=web&cd=&ved=2ahUKEwj0393J_-uCAxXJV0EAHXSNDzoQFnoECBAQAQ&url=https%3A%2F%2Ftraining.cochrane.org%2Fsites%2Ftraining.cochrane.org%2Ffiles%2Fpublic%2Fuploads%2Fresources%2Fdownloadable_resources%2FEnglish%2FCollecting%2520data%2520-%2520form%2520for%2520RCTs%2520only.doc&usg=AOvVaw3jE4L5txeM9LnbpoKk4QgL&opi=89978449

[22] Avancini A, D’Amico F, Tregnago D, Trestini I, Belluomini L, Vincenzi S et al (2021) Nurses’ perspectives on physical activity promotion in cancer patients: a qualitative research. Eur J Oncol Nurs. 10.1016/j.ejon.2021.102061 34763207 10.1016/j.ejon.2021.102061

[23] Avancini A, Tregnago D, Rigatti L, Sartori G, Yang L, Trestini I et al (2020) Factors influencing physical activity in cancer patients during oncological treatments: a qualitative study. Integr Cancer Ther 19:1534735420971365 33349064 10.1177/1534735420971365 PMC7758643

[24] C IJ, Hermens R, Boerboom LWM, Gerritsen WR, van Harten WH, Ottevanger PB (2019) Implementing physical activity programs for patients with cancer in current practice: patients’ experienced barriers and facilitators. J Cancer Surviv 13(5):703–712 31347009 10.1007/s11764-019-00789-3 PMC6828940

[25] Caperchione CM, Sharp P, Phillips JL, Agar M, Liauw W, Harris CA et al (2022) Bridging the gap between attitudes and action: a qualitative exploration of clinician and exercise professional’s perceptions to increase opportunities for exercise counselling and referral in cancer care. Patient Educ Couns 105(7):2489–2496 34823926 10.1016/j.pec.2021.11.002

[26] Cheville AL, Dose AM, Basford JR, Rhudy LM (2012) Insights into the reluctance of patients with late-stage cancer to adopt exercise as a means to reduce their symptoms and improve their function. J Pain Symptom Manage 44(1):84–94 22770487 10.1016/j.jpainsymman.2011.08.009

[27] Coon SKCE (2004) Keep moving: patients with myeloma talk about exercise and fatigue. Oncol Nurs Forum 31:1127–1135 15547635 10.1188/04.ONF.1127-1135

[28] Crandall K, Maguire R, Campbell A, Kearney N (2018) A qualitative study exploring the views, attitudes and beliefs of patients and health professionals towards exercise intervention for people who are surgically treated for lung cancer. Eur J Cancer Care 27(2):e12828 10.1111/ecc.12828 29377387

[29] Emslie C, Whyte F, Campbell A, Mutrie N, Lee L, Ritchie D et al (2007) ‘I wouldn’t have been interested in just sitting round a table talking about cancer’; exploring the experiences of women with breast cancer in a group exercise trial. Health Educ Res 22(6):827–838 17272293 10.1093/her/cyl159

[30] Gokal K, Daley AJ, Madigan CD (2024) Fear of raising the problem without a solution”: a qualitative study of patients’ and healthcare professionals’ views regarding the integration of routine support for physical activity within breast cancer care. Support Care Cancer 32(1):87 38185712 10.1007/s00520-023-08293-2 PMC10771998

[31] Granger CL, Denehy L, Remedios L, Retica S, Phongpagdi P, Hart N et al (2016) Barriers to translation of physical activity into the lung cancer model of care. A qualitative study of clinicians’ perspectives. Ann Am Thorac Soc 13(12):2215–22 27689958 10.1513/AnnalsATS.201607-540OC

[32] Haussmann A, Gabrian M, Ungar N, Jooß S, Wiskemann J, Sieverding M et al (2018) What hinders healthcare professionals in promoting physical activity towards cancer patients? The influencing role of healthcare professionals’ concerns, perceived patient characteristics and perceived structural factors. Eur J Cancer Care 27(4):e12853 10.1111/ecc.12853 29741781

[33] Henriksson A, Arving C, Johansson B, Igelström H, Nordin K (2016) Perceived barriers to and facilitators of being physically active during adjuvant cancer treatment. Patient Educ Couns 99(7):1220–1226 26860549 10.1016/j.pec.2016.01.019

[34] Keogh JW, Patel A, MacLeod RD, Masters J (2014) Perceived barriers and facilitators to physical activity in men with prostate cancer: possible influence of androgen deprivation therapy. Eur J Cancer Care [Engl] 23(2):263–273 10.1111/ecc.12141 24134506

[35] Mikkelsen MK, Nielsen DL, Vinther A, Lund CM, Jarden M (2019) Attitudes towards physical activity and exercise in older patients with advanced cancer during oncological treatment - a qualitative interview study. Eur J Oncol Nurs 41:16–23 31358249 10.1016/j.ejon.2019.04.005

[36] Sheill G, Guinan E, Neill LO, Hevey D, Hussey J (2018) The views of patients with metastatic prostate cancer towards physical activity: a qualitative exploration. Support Care Cancer 26(6):1747–1754 29243168 10.1007/s00520-017-4008-x

[37] Bland KA, Neil-Sztramko SE, Kirkham AA, Bonsignore A, Van Patten CL, McKenzie DC et al (2018) Predictors of attendance to an oncologist-referred exercise program for women with breast cancer. Support Care Cancer 26(9):3297–3306 29651596 10.1007/s00520-018-4180-7

[38] Clark MM, Vickers KS, Hathaway JC, Smith M, Looker SA, Petersen LR et al (2007) Physical activity in patients with advanced-stage cancer actively receiving chemotherapy. J Support Oncol 5(10):487–493 18240671

[39] Courneya KS, Segal RJ, Gelmon K, Reid RD, Mackey JR, Friedenreich CM et al (2008) Predictors of supervised exercise adherence during breast cancer chemotherapy. Med Sci Sports Exerc 40(6):1180–1187 18460985 10.1249/MSS.0b013e318168da45

[40] del Arco A, Martinez Aguirre-Betolaza A, Pérez IM, Malchrowicz-Mośko E, Grajek MK, Krupa-Kotara K et al (2025) Differences in physical activity recommendations, levels of physical activity and main barriers to exercise between Spanish and Polish cancer patients. Healthcare 13(6):598 40150448 10.3390/healthcare13060598 PMC11942116

[41] Felser S, Behrens M, Lampe H, Henze L, Grosse-Thie C, Murua Escobar H et al (2020) Motivation and preferences of cancer patients to perform physical training. Eur J Cancer Care 29(4):e13246 10.1111/ecc.13246 32476203

[42] Frikkel J, Götte M, Beckmann M, Kasper S, Hense J, Teufel M et al (2020) Fatigue, barriers to physical activity and predictors for motivation to exercise in advanced cancer patients. BMC Palliat Care 19(1):43 32234027 10.1186/s12904-020-00542-z PMC7110817

[43] Gho SA, Munro B, Jones SC, Steele JR (2014) Exercise bra discomfort is associated with insufficient exercise levels among Australian women treated for breast cancer. Support Care Cancer 22:721–729 24193222 10.1007/s00520-013-2027-9

[44] Haussmann A, Ungar N, Gabrian M, Tsiouris A, Sieverding M, Wiskemann J et al (2018) Are healthcare professionals being left in the lurch? The role of structural barriers and information resources to promote physical activity to cancer patients. Support Care Cancer 26(12):4087–4096 29934683 10.1007/s00520-018-4279-x

[45] Murnane A, Geary B, Milne D (2012) The exercise programming preferences and activity levels of cancer patients undergoing radiotherapy treatment. Support Care Cancer 20(5):957–962 21523349 10.1007/s00520-011-1167-z

[46] Rogers LQ, Courneya KS, Shah P, Dunnington G, Hopkins-Price P (2007) Exercise stage of change, barriers, expectations, values and preferences among breast cancer patients during treatment: a pilot study. Eur J Cancer Care 16(1):55–66 10.1111/j.1365-2354.2006.00705.x 17227354

[47] Fernandez S, Franklin J, Amlani N, DeMilleVille C, Lawson D, Smith J (2015) Physical activity and cancer: a cross-sectional study on the barriers and facilitators to exercise during cancer treatment. Can Oncol Nurs J 25(1):37–48 26642493 10.5737/236880762513742

[48] Mazzoni A-S, Carlsson M, Berntsen S, Nordin K, Demmelmaier I (2019) “Finding my own motivation” — a mixed methods study of exercise and behaviour change support during oncological treatment. Int J Behav Med 26(5):499–511 31441015 10.1007/s12529-019-09809-z PMC6785591

[49] Murphy K, Kehoe B, Denieffe S, McGrath A, Hacking D, Fairman CM et al (2024) ‘Just because I have prostate cancer doesn’t mean that I can’t do things’ – men’s experiences of the acceptability of an exercise intervention for prostate cancer during treatment. BMC Cancer 24(1):949 39095735 10.1186/s12885-024-12687-8 PMC11297682

[50] Gildea GC, Spence RR, Jones TL, Turner JC, Macdonald ER, Hayes SC et al (2023) Barriers, facilitators, perceptions and preferences influencing physical activity participation, and the similarities and differences between cancer types and treatment stages - a systematic rapid review. Prev Med Rep 34:102255 37273528 10.1016/j.pmedr.2023.102255 PMC10236469

[51] Clifford BK, Mizrahi D, Sandler CX, Barry BK, Simar D, Wakefield CE et al (2018) Barriers and facilitators of exercise experienced by cancer survivors: a mixed methods systematic review. Support Care Cancer 26(3):685–700 29185105 10.1007/s00520-017-3964-5

[52] Van Dijck S, De Groef A, Kothari J, Dams L, Haenen V, Roussel N et al (2023) Barriers and facilitators to physical activity in cancer survivors with pain: a systematic review. Support Care Cancer 31(12):668 37922014 10.1007/s00520-023-08141-3

