# Supplementary material for: A systematic review of patient and healthcare professional perceptions of the barriers and facilitators to embedding exercise in the adjuvant cancer treatment pathway
Source: Support Care Cancer. 2026 Mar 18;34(4):342. doi: 10.1007/s00520-026-10553-w (PMC12999592; doi:10.1007/s00520-026-10553-w)
Supplement: Supplementary file 2 — (DOCX 15.7 KB) [file 520_2026_10553_MOESM2_ESM.docx]

**Online Resource 2:** Example Search Strategy/Terms used across Databases

Refined by: Publication Date, English Language, All Adult

Databases searched: CINAHL Plus, Academic Search Premier, Business Source Premier, Ebook collection (EBSCOHost), Education Research Complete, ERIC, MEDLINE, APA PsycArticles, PsycINFO, Regional Business News, SPORT Discus and Cochrane

1. Cancer patient* OR oncology patients OR patients with cancer AND exercise OR physical activity AND perceptions
2. Cancer patient* OR oncology patients OR patients with cancer AND exercise OR physical activity AND perceptions OR barriers
3. Cancer patient* OR oncology patients OR patients with cancer AND exercise OR physical activity AND perceptions OR barriers OR facilitators OR attitudes OR beliefs OR experience* OR lived experience*
4. Cancer patient* OR oncology patients OR patients with cancer OR cancer treatment OR chemo* OR radiotherapy OR treatment pathway OR malignant neoplasm OR malign* OR neoplasm OR adjuvant OR adjuvant treatment OR adjuvant cancer treatment OR oncology care AND exercise OR embedding OR embedding exercise OR exercise therap* OR exercise intervention* OR physical activity OR rehabilitation OR rehab* AND perceptions OR barriers OR facilitators OR attitudes OR beliefs OR experience* OR lived experience* OR quality of life OR perioperative outcomes
5. Cancer patient* OR oncology patients OR patients with cancer OR cancer treatment OR chemo* OR radiotherapy OR treatment pathway OR malignant neoplasm OR malign* OR neoplasm OR adjuvant OR adjuvant treatment OR adjuvant cancer treatment OR oncology care AND exercise OR embedding OR embedding exercise OR exercise therap* OR exercise intervention* OR physical activity OR rehabilitation OR rehab* AND perceptions OR barriers OR facilitators OR attitudes OR beliefs OR experience* OR lived experience* OR quality of life OR perioperative outcomes
6. Healthcare professional OR healthcare practitioner AND exercise OR physical activity
7. Healthcare professional OR healthcare practitioner OR exercise professional OR exercise physiologist OR oncology provider AND exercise OR physical activity AND perceptions
8. Healthcare professional OR healthcare practitioner OR exercise professional OR exercise physiologist OR oncology provider AND exercise OR physical activity OR rehabilitation OR rehab* AND perceptions OR barriers OR facilitators OR attitudes OR beliefs OR experience* OR lived experience
