# Supplementary material for: A systematic review of patient and healthcare professional perceptions of the barriers and facilitators to embedding exercise in the adjuvant cancer treatment pathway
Source: Support Care Cancer. 2026 Mar 18;34(4):342. doi: 10.1007/s00520-026-10553-w (PMC12999592; doi:10.1007/s00520-026-10553-w)
Supplement: Supplementary file 3 — (DOCX 20.2 KB) [file 520_2026_10553_MOESM3_ESM.docx]

**Online Resource 3:** Quality Appraisal of Included Studies adapted from the Critical Appraisal Skills Programme (CASP) Tool (n=29)

| **Study** | **Study design** | **Appropriateness of study design** | **Risk of bias** | **Statistical issues** | **Quality of reporting** | **Quality of intervention** | **Generalisability** |
| --- | --- | --- | --- | --- | --- | --- | --- |
| Avancini et al (2021) [22] | Qualitative | ✓ | 🗶 | N/A | ✓ | ✓ | ✓ |
| Avancini et al (2020) [23] | Qualitative | ✓ | 🗶 | N/A | 🗶 | ✓ | 🗶 |
| Bland et al (2018) [37] | Quantitative | 🗶 | ✓ | 🗶 | 🗶 | ✓ | ✓ |
| Caperchione et al (2021) [25] | Qualitative | ✓ | ✓ | N/A | ✓ | ✓ | ✓ |
| Cheville et al (2012) [26] | Qualitative | ✓ | 🗶 | ✓ | ✓ | 🗶 | 🗶 |
| Clark et al (2007) [38] | Quantitative |  | ✓ |  | ✓ | ✓ | ✓ |
| Coon and Coleman (2004) [27] | Qualitative | ✓ | 🗶 | ✓ | ✓ | ✓ | 🗶 |
| Courneya at al (2008) [39] | Quantitative | ✓ | ✓ | ✓ | ✓ | ✓ | ✓ |
| Crandall et al (2018) [28] | Qualitative | ✓ | ✓ | N/A | 🗶 | ✓ | 🗶 |
| Del Arco et al (2025) [40] | Quantitative | ✓ | ✓ | ✓ | ✓ | ✓ | 🗶 |
| Emslie et al (2007) [29] | Qualitative | ✓ | ✓ | N/A | ✓ | ✓ | ✓ |
| Felser et al (2019) [41] | Quantitative | ✓ | 🗶 | ✓ | 🗶 | 🗶 | 🗶 |
| Fernandez et al (2015) [47] | Mixed Methods | ✓ | 🗶 | ✓ | ✓ | 🗶 | 🗶 |
| Frikkel et al (2020) [42] | Quantitative | ✓ | ✓ | 🗶 | ✓ | ✓ | 🗶 |
| Gho et al (2013) [43] | Quantitative | ✓ | 🗶 | ✓ | 🗶 | 🗶 | 🗶 |
| Gokal et al (2024) [30] | Qualitative | ✓ | ✓ | N/A | 🗶 | ✓ | ✓ |
| Granger et al (2016) [31] | Qualitative | ✓ | 🗶 | N/A | 🗶 | ✓ | 🗶 |
| Haussman et al (2018) [32] | Qualitative | ✓ | ✓ | N/A | ✓ | ✓ | ✓ |
| Haussman et al (2018) [44] | Quantitative | ✓ | ✓ | ✓ | 🗶 | ✓ | 🗶 |
| Henriksson et al (2016) [33] | Qualitative | ✓ | 🗶 | N/A | ✓ | 🗶 | ✓ |
| Ijsbrandy et al (2019) [24] | Qualitative | ✓ | ✓ | N/A | 🗶 | ✓ | 🗶 |
| Keogh et al (2014) [34] | Qualitative | ✓ | ✓ | N/A | ✓ | 🗶 | ✓ |
| Mazzoni et al (2019) [48] | Mixed methods | ✓ | 🗶 | ✓ | ✓ | ✓ | ✓ |
| Midtgaard et al (2009) [14] | Quantitative | ✓. | ✓ | ✓ | ✓ | 🗶 | ✓ |
| Mikkelson et al (2019) [35] | Qualitative | ✓ | ✓ | 🗶 | 🗶 | 🗶 | ✓ |
| Murnane et al (2010) [45] | Quantitative | ✓ | 🗶 | ✓ | ✓ | 🗶 | ✓ |
| Murphy et al (2024) [49] | Qualitative | ✓ | ✓ | N/A | 🗶 | ✓ | ✓ |
| Rogers et al (2007) [46] | Quantitative | ✓ | ✓ | ✓ | 🗶 | 🗶 | ✓ |
| Sheill et al (2017) [36] | Qualitative | ✓ | ✓ | 🗶 | ✓ | 🗶 | 🗶 |
| Notes: N/A, Not Applicable | | | | | | | |
