# Supplementary material for: A systematic review of patient and healthcare professional perceptions of the barriers and facilitators to embedding exercise in the adjuvant cancer treatment pathway
Source: Support Care Cancer. 2026 Mar 18;34(4):342. doi: 10.1007/s00520-026-10553-w (PMC12999592; doi:10.1007/s00520-026-10553-w)
Supplement: Supplementary file 4 — (DOCX 21.4 KB) [file 520_2026_10553_MOESM4_ESM.docx]

**Online Resource 4:** Patient Barriers and Facilitators Mapped to the Capability-Opportunity-Motivation-Behaviour (COM-B) Model and Theoretical Domains Framework (TDF)

| **Themes** | **References** |
| --- | --- |
| **Capability-related barriers** | |
| **C1. Physical capability** | |
| **C1.1 Exercise Intervention - Lack of knowledge (TDF domain ‘skills’)** | |
| C1.1.1 Lack of information regarding physical activity | 24, 14, 23, 30, 47 |
| C1.1.2 Lack of communication from HCP’s | 24, 23, 30, 35, 38 |
| C1.1.3 Highly structured, non-daily activity orientated exercise | 26 |
| C1.1.4 Inaccessibility of qualified contact persons | 24 |
| **C1.2 Impact of Cancer – Treatment related symptoms (TDF domain ‘knowledge’)** | |
| C1.2.1 Treatment side effects | 14, 23, 26, 27, 36, 43, 41 |
| C1.2.2 Physical symptoms from treatment | 14, 27, 29, 33, 35, 36, 38, 45, 43, 42, 47, 40 |
| **C2. Psychological capability** | |
| **C2.1 Impact of Cancer – Cancer site (TDF domain ‘knowledge’)** | |
| C2.1.1 Disease and progression | 14, 27, 36 |
| C2.1.2 Impact on pre-existing co-morbidities | 33, 46, 42 |
| C2.1.3 Cancer site specific symptoms | 14, 29, 43 |
| **Opportunity-related barriers** | |
| **O1. Physical opportunity** | |
| **O1.1 Exercise Intervention - Social barriers and connection (TDF domain ‘environmental context and resources’)** | |
| O1.1.1 Waiting lists | 24 |
| O1.1.2 Inconvenient exercise schedule | 14, 46 |
| **O1.2 Setting – Environment (TDF domain ‘environmental context and resources’)** | |
| O1.2.1 Weather seasonality | 23, 26, 27, 35, 40 |
| O1.2.2 Place and time | 24, 46, 45 |
| O1.2.3 Travel distance | 24, 49, 48, 27, 28 |
| O1.2.4 Cost | 28, 46, 49 |
| O1.2.5 Home environment | 27, 35 |
| **O1.3 Setting – Organisational resources (TDF domain ‘environmental context and resources’)** | |
| O1.3.1 No paid transport | 42 |
| O1.3.2 Economic hindrances | 14, 37, 40 |
| O1.3.3 Lack of equipment | 46 |
| O1.3.4 Laws and governance | 24, 30 |
| **O2. Social opportunity** | |
| **O2.1 Exercise Intervention - Social barriers and connection (TDF domain ‘social influences’)** | |
| O2.1.1 Time and other commitments | 14, 27, 28, 35, 46, 42, 48, 40 |
| O2.1.2 Protective family | 28, 46 |
| O2.1.3 Difficulty exercising independently | 14, 33, 36, 49 |
| O2.1.4 Unsure what ‘everyone else’ is doing | 36, 49 |
| O2.1.5 Life before cancer and social environment | 27 |
| O2.1.7 Lack of childcare | 27 |
| O2.2.8 Feeling ‘vulnerable’ in group settings | 24, 33, 49 |
| **Motivation-related barriers** | |
| **M1. Reflective motivation** | |
| **M1.1 Exercise Intervention - Beliefs around capability (TDF domain ‘beliefs about capabilities)** | |
| M1.1.1 Fear of exercise being harmful | 14, 26, 27, 28, 30, 35, 49 |
| M1.1.2 Non-exercise people | 26, 33, 42 |
| M1.1.3 Exercise is an additional activity | 26 |
| M1.1.4 Female role in the family | 29, 37 |
| **M1.2 Setting – Perceptions of delivery (TDF domain ‘social identity’)** | |
| M1.2.1 Age | 33, 35, 41, 40 |
| **M1.3 Impact of Cancer – Emotional wellbeing and self-belief (TDF domain ‘optimism’)** | |
| M1.3.1 Readiness and loss of confidence | 24, 28, 29, 30, 37, 41, 47, 49 |
| M1.3.2 Low mood and motivation | 28, 33, 36, 46, 42 |
| M1.3.3 Lack of interest in exercise | 27, 46, 42, 47 |
| **M2. Automatic motivation** | |
| **M2.1 Exercise Intervention - Lack of knowledge (TDF domain ‘emotion’)** | |
| M2.1.1 Lack of enjoyment in the exercise provided | 46, 48 |
| **Capability-related facilitators** | |
| **C2. Psychological capability** | |
| **C2.1 Exercise Intervention – Self-belief and capability (TDF domain ‘knowledge’)** | |
| C2.1.1 Previous positive experience with exercise | 14, 23, 26, 27, 42, 41, 47 |
| **Opportunity-related facilitators** | |
| **O.1 Physical opportunity** | |
| **O1.1 Setting – Accessibility and resources (TDF domain ‘environmental context and resources’)** | |
| O1.1.1 Cost | 41 |
| O1.1.2 Travel | 24, 30, 36 |
| O1.1.3 Participation in research | 27 |
| **O1.2 Impact of Cancer – Alleviate symptom burden (TDF domain ‘environmental context and resources’)** | |
| O1.2.1 Accessible toileting facilities | 41 |
| **O.2 Social opportunity** | |
| **O2.1 Intervention – Self-belief and capability (TDF domain ‘social influences’)** | |
| O2.1.1 Positive promotion from family and HCPs | 26, 29, 30, 35, 36, 38, 37, 42, 47, 48, 40 |
| O2.1.2 Exercise prescription | 37, 47 |
| **O2.2 Setting – Individualised care (TDF domain ‘social influences’)** | |
| O2.2.1 Individualised programming | 27, 28, 30, 33, 35, 45, 41, 48, 40 |
| O2.2.2 Flexible training times | 30, 37, 41 |
| O2.2.3 Flexible settings for delivery | 24, 23, 25, 28, 30, 35, 45, 47, 48 |
| **O2.3 Setting – Credible sources (TDF domain ‘social influences’)** | |
| O2.3.1 Credible exercise practitioner | 23, 26, 28, 30, 35, 36, 41, 48 |
| O2.3.2 Guidance from a medical professional | 26, 34, 42 |
| **O2.4 Setting – Social support and connection (TDF domain ‘social influences’)** | |
| O2.4.1 Group exercise sessions | 30, 35, 41, 48 |
| O2.4.2 Solidarity and friendships | 24, 23, 28, 29, 34 |
| O2.4.3 Family involvement | 23, 28, 34 |
| **Motivation-related facilitators** | |
| **M1. Reflective motivation** | |
| **M1.1 Exercise Intervention – Self-belief and capability (TDF domain ‘beliefs about capabilities’)** | |
| M1.1.1 Motivation to participate | 24, 27, 34, 42, 41, 48 |
| M1.1.3 Benefitting quality of life | 26, 37 |
| **M1.2 Impact of Cancer – Alleviate symptom burden (TDF domain ‘goals’)** | |
| M1.2.1 Exercise is part of treatment plan | 23, 27, 34, 35 |
| M1.2.2 Satisfaction | 26, 27, 28, 48 |
| M1.2.3 Improvement in energy levels | 27, 35, 48 |
| **M1.3 Impact of Cancer – Time-efficient pathway (TDF domain ‘beliefs about capabilities’)** | |
| M1.3.1 Programming throu43ut treatment | 23, 33, 35, 46, 45, 47 |
| M1.3.2 Resources matching stage of change | 27, 46 |
| **M1.4 Exercise Intervention – Self-belief and capability (TDF domain ‘beliefs about capabilities’)** | |
| M1.4.1 Gender | 14, 29 |
| M1.4.2 Socioeconomic status | 37 |
