# Supplementary material for: A systematic review of patient and healthcare professional perceptions of the barriers and facilitators to embedding exercise in the adjuvant cancer treatment pathway
Source: Support Care Cancer. 2026 Mar 18;34(4):342. doi: 10.1007/s00520-026-10553-w (PMC12999592; doi:10.1007/s00520-026-10553-w)
Supplement: Supplementary file 5 — (DOCX 17.9 KB) [file 520_2026_10553_MOESM5_ESM.docx]

**Online Resource 5:** Healthcare Professional (HCP) Barriers and Facilitators Mapped to the Capability-Opportunity-Motivation-Behaviour (COM-B) Model and Theoretical Domains Framework (TDF)

| **Themes** | **References** |
| --- | --- |
| **Capability-related barriers** | |
| **C1. Physical capability** | |
| **C1.1 Exercise Intervention – Lack of knowledge (TDF domain ‘skills’)** | |
| C1.1.1 Lack of information for medical staff | 24, 22, 28, 30, 31, 32, 44 |
| C1.1.2 Lack of information for cancer patients | 44 |
| C1.1.3 Lack of specific guidelines | 30, 31, 44 |
| **C2. Psychological capability** | |
| **C2.1 Exercise Intervention – Beliefs and communication skills (TDF domain ‘skills’)** | |
| C2.1.1 Lack of time during consultations | 44 |
| C2.1.2 Reactive discussions | 25, 30 |
| C2.1.3 Skills in behaviour change | 30, 31, 32 |
| **Opportunity-related barriers** | |
| **O1. Physical opportunity** | |
| **O1.1 Setting – Availability of resources (TDF domain ‘environmental context and resources)** | |
| O1.1.1 Lack of programme availability | 24, 22, 28, 30, 31, 32, 44 |
| O1.1.2 Inconsistency referral process | 25, 44 |
| O1.1.3 Limited space and capacity in hospitals | 25, 31 |
| O1.1.4 Lack of expert person | 22, 28, 31, 44 |
| O1.1.5 Lack of time during shift | 22, 30, 31 |
| O1.1.6 Lack of individualised care | 24 |
| **Motivation-related barriers** | |
| **M1. Reflective motivation** | |
| **M1.1 Impact of Cancer – Belief about capability (TDF domain ‘optimism’)** | |
| M1.1.1 Perception of patient ability | 22, 31, 32 |
| M1.1.2 Fear of causing psychological difficulty | 32 |
| **M2. Automatic motivation** | |
| **M2.1 Impact of Cancer – Symptom focus (TDF domain ‘reinforcement’)** | |
| M2.1.1 Medical focus during consultations | 22, 31 |
| M2.1.2 Symptoms associated with specific cancer site | 25, 31 |
| **Capability-related facilitators** | |
| **C1. Physical capability** | |
| **C1.1 Exercise Intervention – Training and education (TDF domain ‘skills’)** | |
| C1.1.1 Communication skills | 22, 25, 30 |
| C1.1.2 Specific educational resources and guidance | 22, 25, 30, 31, 32 |
| **C2. Psychological capability** | |
| **C2.1 Impact of cancer – Culture shift (TDF domain ‘behavioural regulation’)** | |
| C2.1.1 Making physical activity a priority in consultations | 30, 31 |
| C2.1.2 Escapism from cancer diagnosis and treatment | 30 |
| C2.1.3 Gender and participation | 44 |
| **Opportunity-related facilitators** | |
| **O.1 Physical opportunity** | |
| **O1.1 Setting – Credible practitioner (TDF domain ‘environmental context and resources’)** | |
| O1.1.1 Instructor skill and credibility | 25, 31 |
| **O1.2 Setting – Individualised care with resources (TDF domain ‘environmental context and resources’)** | |
| O1.2.1 Written information and resources | 22, 25, 30, 31, 44 |
| O1.2.2 Physical activity prescription | 22, 28, 31 |
| O1.2.3 Availability of space | 22, 30, 32 |
| **O.2 Social opportunity** | |
| **O2.1 Setting – Sense of safety (TDF domain ‘social influences’)** | |
| O2.1.1 Teamwork | 22 |
| **O2.2 Impact of cancer – Time-efficient pathway (TDF domain ‘social influences’)** | |
| O2.2.1 Delivery of information compatible with treatment | 25, 30, 31, 32 |
| O2.2.2 Frequency of conversations | 30, 31 |
| O2.2.3 Direct referral (not GP only | 25, 31 |
| **Motivation-related facilitators** | |
| **M1. Reflective motivation** | |
| **M1.1 Exercise Intervention – Understanding patient capability (TDF domain ‘beliefs about consequences’)** | |
| M1.1.1 Understanding patient headspace | 25, 30, 32 |
| M1.1.2 Witnessing benefits | 31, 25 |
